# Comparison of Solubilization Capacity of Resveratrol in Sodium 3**α**,12**α**-Dihydroxy-7-oxo-5**β**-cholanoate and Sodium Dodecyl Sulfate

**DOI:** 10.1155/2014/265953

**Published:** 2014-01-29

**Authors:** Jelena Cvejić, Mihalj Poša, Ana Sebenji, Milica Atanacković

**Affiliations:** Department of Pharmacy, Faculty of Medicine, Hajduk Veljkova 3, University of Novi Sad, 21000 Novi Sad, Serbia

## Abstract

In this study we investigated resveratrol (*trans*-3,5,4′-trihydroxystilbene) solubilization with sodium 3**α**,12**α**-dihydroxy-7-oxo-5**β**-cholanoate (S7-OD) and sodium dodecyl sulfate (SDS). The investigation was aimed at determining whether large spherical micelles (SDS) or small longitudinal micelles (S7-OD) are more convenient for incorporation of resveratrol. Also, we studied resveratrol behavior in mixed micelles with mentioned surfactants using spectroflourimetric method as well as the effects of sodium chloride and urea on resveratrol solubilization capacity in the applied surfactants. Resveratrol solubilization curve was different in the investigated surfactants. Resveratrol solubilization curve for sodium 3**α**,12**α**-dihydroxy-7-oxo-5**β**-cholanoate at concentration 0.9 CMC reached saturation level of 60% dissolved resveratrol. The curve for sodium dodecyl sulfate was linear within the whole range of the investigated concentration; resveratrol solubilization rate reached 13% at 2 CMC. In S7-OD, NaCl increased capacity of resveratrol solubilization up to 1.4 CMC surfactant concentration, whilst maximum level of dissolved resveratrol (90%) was observed at 0.9 CMC. In SDS, NaCl decreased resveratrol solubilization capacity. Urea reduced resveratrol solubilization rate in sodium 3**α**,12**α**-dihydroxy-7-oxo-5**β**-cholanoate, whereas it had inverse effect in sodium dodecyl sulfate. The obtained results strongly suggest that structure, that is, shape, of the surfactant micelles significantly affects their capacity of resveratrol solubilization. Also, presence of NaCl and urea influences solubilization capacities of investigated surfactants.

## 1. Introduction

Increase of surfactant (amphiphilic molecules) concentration results in formation of aggregates, that is, micelles. Above the critical micelle concentration (CMC) increase of surfactant concentration is associated with constant concentration of free amphiphilic molecules (ions), whereas level of micellar phase (association colloid) is elevated. Micelles are characterized by hydrophobic inner core and hydrophilic outer surface [1–4]. Thus, micelles are applicable to improve solubility of hydrophobic molecules within the hydrophobic cage of the micelle [5, 6].

**Figure 1:**
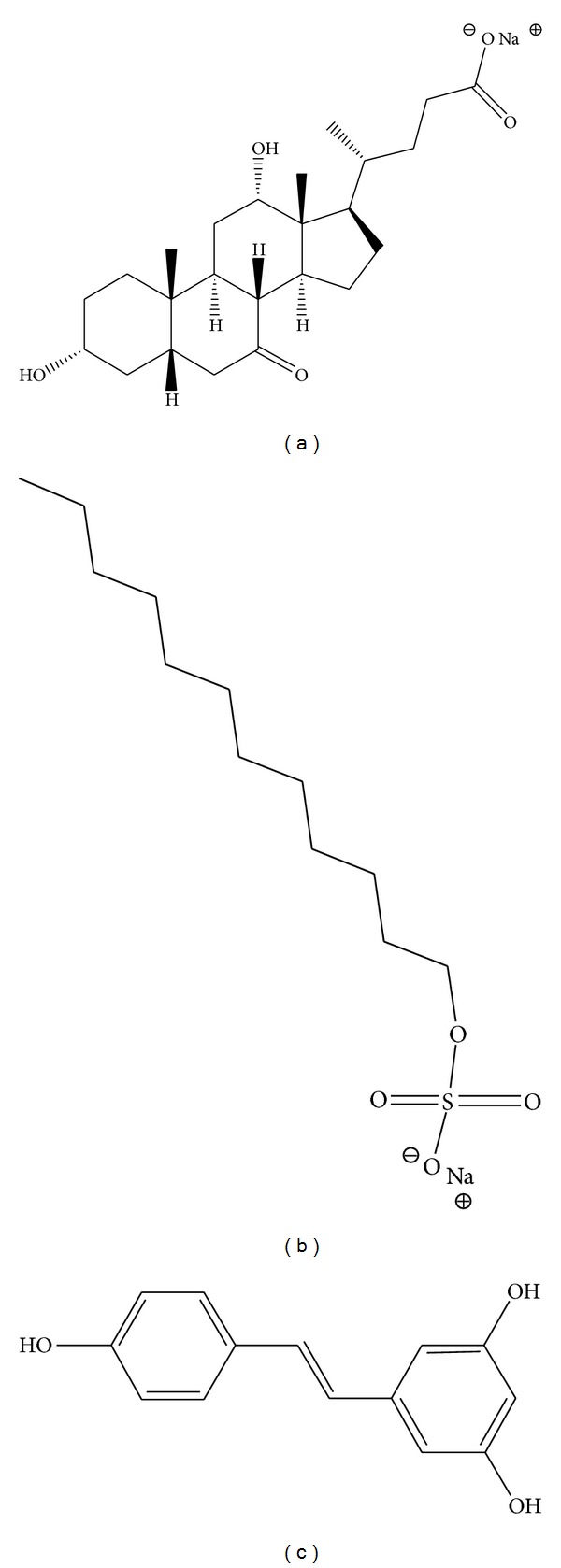
Analyzed surface-active substances (a) sodium 3*α*,12*α*-dihydroxy-7-oxo-5*β*-cholanoate (sodium-7-oxo-deoxycholate, S7-OD), (b) sodium dodecyl sulphate (SDS), and (c) resveratrol (*trans*-3,5,4′-trihydroxystilbene).

Resveratrol (*trans*-3,5,4′-trihydroxystilbene; Figure 1) is a natural polyphenol with well-established antioxidative activity and a range of pharmacological applications. However, under physiological conditions it is poorly water-soluble (pH 6: 0.22 nmol/L; pH 8: 0.23 nmol/L; pH 10.8: 0.67 nmol/L) [7–12].

Sodium salt of 3*α*,12*α*-dihydroxy-7-oxo-5*β*-cholanoic acid (S7-OD) is oxo derivative of cholic acid (Figure 1). The replacement of the C7 OH group of cholic acid with an oxo group results in reduced hydrophobicity of *β*-side of the steroid skeleton in newly formed oxo derivative. Presence of the oxo group in the steroid skeleton reduces affinity of bile salts to form micelles (increases their CMC value) and at the same time decreases their membrane toxicity [13]. Oxo derivatives of cholic acid express a range of pharmacological effects associated with their amphiphilicity [14–16].

Sodium dodecyl sulfate (SDS) is an aliphatic surfactant (Figure 1). SDS forms spherical micelles with a hydrophobic core [5, 17]. In pharmacy it is used as a component of some aspirin formulations aimed at increasing their solubility. SDS is used in numerous cosmetic products; however, there are reports on its negative effects and possible skin damage [18].

The aim of this study was to determine which of the two applied surfactants has better capacity to solubilize resveratrol, that is, whether large spherical micelles (SDS) or small longitudinal micelles (S7-OD) are more appropriate for resveratrol incorporation. A further objective of the research is to investigate effects of sodium chloride and urea on resveratrol solubilization. Namely, application of urea and NaCl results in modeling real biological fluid, such as plasma. Possible promoter effect of NaCl and urea on resveratrol solubilization in the investigated surfactants may enable application of smaller amounts of surface-active substance and yielding the same solubilization effect, thus reducing potential membrane toxic capacity of the surfactant.

## 2. Materials and Methods

### 2.1. Materials

Cholic acid (Sigma, New Zealand, 98%) was used for the synthesis of 3*α*,12*α*-dihydroxy-7-oxo-5*β*-cholanoic acid according to the procedure by Tullar [19]. Urea and NaCl were purchased from Merck (analytical reagent grade). Standard transresveratrol (*trans*-3,5,4′-trihydroxystilbene) was provided by Sigma (Germany). Methanol was obtained from JT Baker (Holland). All solvents were of HPLC grade.

### 2.2. Determination of Resveratrol Solubilization

Resveratrol (4 mM) solubilization was determined according to Atanacković et al. [20] at 25°C. In the experiment investigating effects of NaCl and urea, each buffered (pH 8.00 phosphate buffer) solution contained concentrations of 0.3 M and 2 M NaCl and urea, respectively. Each measurement was repeated five times.

### 2.3. Analytical HPLC Procedure

Resveratrol was determined according to modified Trela and Waterhouse (1996) HPLC method for determination of resveratrol [21].

Agilent 1100 series with binary pump, autosampler, and a diode array UV/Vis detector was used. Analyses were conducted with Zorbax SB-C18 4.6 × 150 mm, 5 *μ*m column, and with Zorbax SB-C18 4.6 × 12.5 mm, 5 *μ*m precolumn. The eluents were (A) water and (B) methanol. Gradient elution was performed by using 50% B at 0 to 100% B at 8 min. Run time was 8.5 min. Injection volume was 5 *μ*L and the column was thermostatically controlled to maintain a temperature of 37°C. The eluent was monitored at 305 and UV spectra of the eluent peaks were obtained.

Quantitation was accomplished by comparison with a standard response curve prepared from solutions of resveratrol in methanol.

Results of the analysis were expressed as percentage of solubilized resveratrol.

### 2.4. The Fluorescence Study

Measurements were performed by the Cary Eclipse Fluorescence Spectrophotometer. The wavelength of the excitation light was 330 nm and the emission wavelength was 360 nm. Temperature was changed from 0°C to 50°C in intervals of 10°C.

The 3D models (energetically most favorable) of bile acids molecules are generated according to the MOPAC protocol (ChemBio3D Ultra 11.0).

## 3. Results and Discussion

### 3.1. Solubilization of Resveratrol

**Figure 2:**
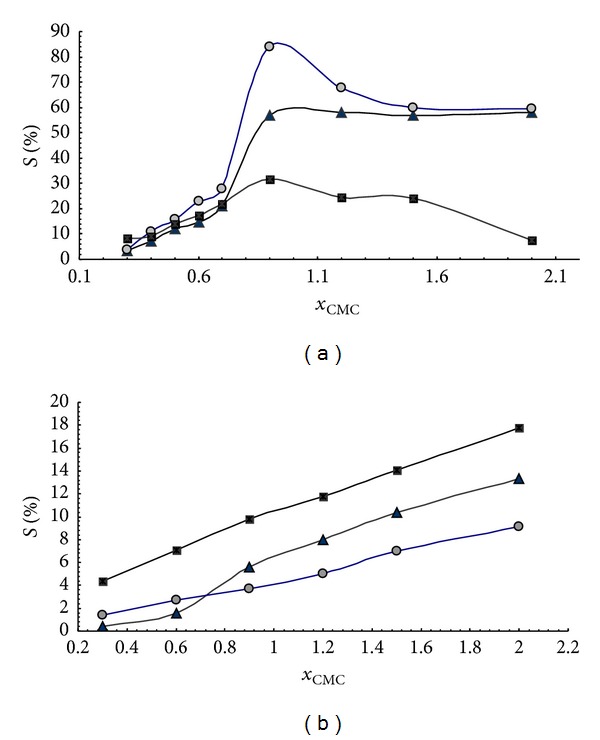
Solubilization curves (median values) resveratrol (4 mM) in presence of (a) sodium 3*α*,12*α*-dihydroxy-7-oxo-5*β*-cholanoate and (b) sodium dodecyl sulfate (black triangle: surfactant, pH 8.00 phosphate buffer; grey circle: surfactant + 0.3 M NaCl, pH 8.00 phosphate buffer; black square: surfactant + 2 M urea, pH 8.00 phosphate buffer.

**Figure 3:**
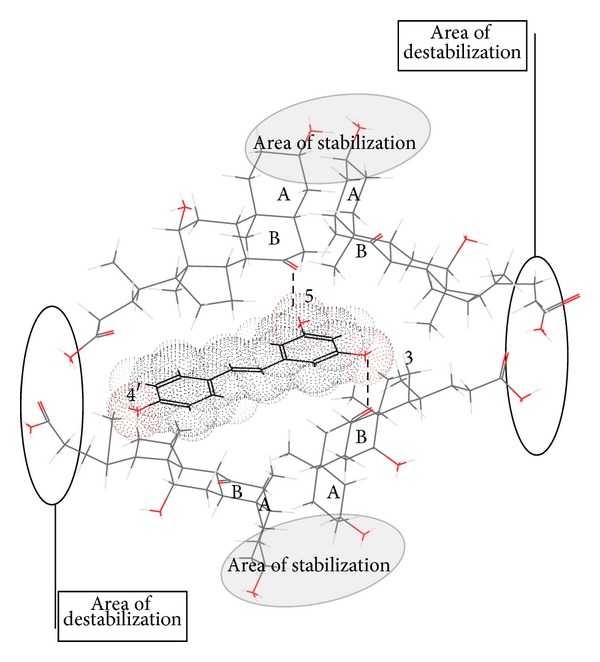
Mixed micelle of resveratrol and sodium 3*α*,12*α*-dihydroxy-7-oxo-5*β*-cholanoate (dotted lines depict hydrogen bonds).

**Figure 4:**
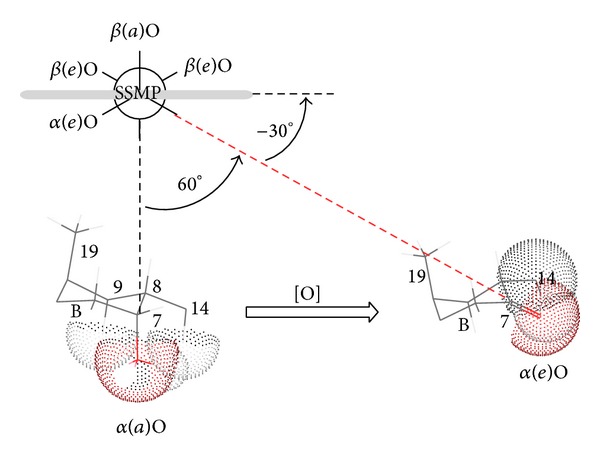
Change in oxygen-core orientation during oxidation.

Solubilization curve (Figure 2) indicates differences in resveratrol-solubilization behavior of sodium salts of 3*α*,12*α*-dihydroxy-7-oxo-5*β*-cholanoic acid (S7-OD) and sodium dodecyl sulfate (SDS). Based on critical micellar concentration (CMC) values one might expect that more hydrophobic SDS (CMC = 8.00 mM) [5] has better resveratrol solubilization capacity than S7-OD (CMC = 43 mM) [22]. However, experiments indicated the contrary. Namely, resveratrol solubilization curve in the presence of sodium 3*α*,12*α*-dihydroxy-7-oxo-5*β*-cholanoate yields saturation rate of 60% at 0.9 CMC. In the presence of sodium dodecyl-sulfate resveratrol solubilization curve is linear and, even at 2 CMC, micellar phase yields solubilization rate of 13%. This difference can be attributed to the micelle type of the investigated surfactants. Sodium 3*α*,12*α*-dihydroxy-7-oxo-5*β*-cholanoate is characterized by longitudinal micelles with aggregation numbers 2–8, whereas dodecyl sulfate forms spherical micelles of up to 100 monomer units [23]. The symmetry of the probe molecule (planar, electron cloud: longitudinal) indicates that resveratrol fits better into the longitudinal micelle of S7-OD then into the spherical SDS micelle. Some former research of Atanacković et al. revealed that sodium salts of bile acids containing oxo group solubilize resveratrol more efficiently than bile acids containing *α*-axial OH groups [20]. Namely, hydrophobic micellar interior of the sodium 3*α*,12*α*-dihydroxy-7-oxo-5*β*-cholanoate micelle, between the steroid skeleton of bile acids and resveratrol, besides the hydrophobic interactions, contains additional hydrogen bonds between C7 oxo group and OH group of the guest molecule (Figure 3). This additional interaction is possible as oxidation of C7 OH group of cholic acid results in formation of oxo group whose oxygen atom is shifted for 60°—in an appropriate Newman projection formula—in relation to the initial *α* axial OH group (Figure 4). That implies that oxygen atom of the oxo group is shifted towards the *β*-side of the steroid skeleton (forming a −30° angle with the mean plane of the steroid skeleton of SSMP), that is, towards the hydrophobic interior of mixed micelle.

NaCl increases capacity of S7-OD to solubilize resveratrol below and around the CMC value. Solubilization curve reached the peak at 0.9 CMC, whilst beyond 1.5 CMC the solubilization curve does not differ from that without NaCl. Effects of NaCl around CMC result from the “salting-out effect” of sodium chloride that has promoter effect on desolvatization of sodium 3*α*,12*α*-dihydroxy-7-oxo-5*β*-cholanoate, which decreases its CMC value and induces aggregate formation at lower concentrations [22–25]. Furthermore, NaCl adsorbs at the surface of the mixed micelle and increases its stability by neutralizing electric charge of carboxylate groups of the side chain of bile acid [22] (area of destabilization of the mixed micelle) and hence reducing repulsive interactions between 3*α*,12*α*-dihydroxy-7-oxo-5*β*-cholanoate in the aggregate (Figure 3). NMR relaxation research of Poša et al. revealed that, beyond CMC value, at the 75 mM, S7-OD forms secondary micelles (primary micelles bonded by hydrogen bonds) [22]. It is well established that NaCl dehydratizes the OH groups on the outer side of mixed (primary) micelle, thus enhancing formation of hydrogen bonds, that is, secondary micelles [23].

**Figure 5:**
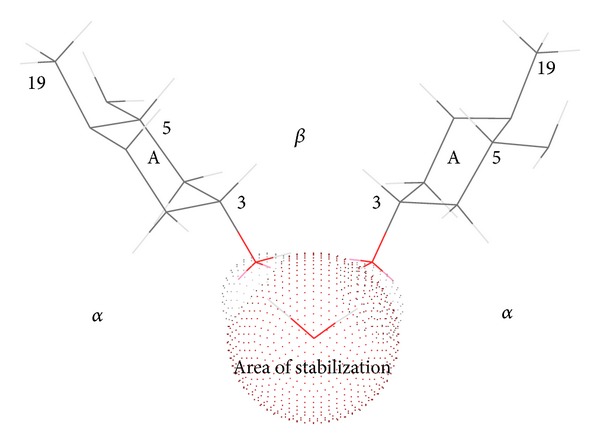
Area of stabilization of mixed micelle (a ring of 3*α*,12*α*-dihydroxy-7-oxo-5*β*-cholanoic acid).

However, in secondary micelles (probably due to hydrogen bonds between adjacent primary micelles), the 3*α*,12*α*-dihydroxy-7-oxo-5*β*-cholanoate of the adjacent primary micelles are shifted toward each other, resulting in disturbance of hydrophobic interior of the primary micelle. Namely, increasing the distance between primary micelle monomer units (S7-OD) prevents further stabilization of the hydrophobic cage by hydrogen bonds (via the water) between equatorial C3 OH groups of bile acid (Figures 3 and 5). As a result, the hydrophobic cage undergoes hydration, thus reducing hydrophobic interaction between steroid skeleton of bile acid and resveratrol.

Urea significantly reduces resveratrol solubilization capacity of 3*α*,12*α*-dihydroxy-7-oxo-5*β*-cholanoate as a result of complementary bonding of urea via the hydrogen bond to the oxo group of the analyzed bile acid. At concentrations below CMC bile acids form aggregates with most probable aggregation number 2. Dimeric micelles are characterized by a “fiord” at their surface (fissure at the joint of monomer units, a portal to the hydrophobic cage) [4]. In keto derivatives of bile acids oxo-groups are located in the fiord [25], where they form hydrogen bond with OH group of resveratrol [20]. However, amino group of urea competes with formation of hydrogen bond resulting in destabilization of mixed micelle. On the other hand, at concentrations beyond CMC, urea exhibits promoter effect on formation of secondary micelles [22], which results in reduced hydrophobicity of the primary micelle (similarly to NaCl).

NaCl slightly increases resveratrol solubilization capacity of SDS at levels up to 0.6 CMC, probably as a result of reduced repulsive interactions between negatively charged polar heads of SDA at micellar surface caused by sodium ions adsorption. Beyond 0.6 CMC sodium chloride exhibits contrary effect. The possible explanation lies in Tanford scheme of water penetration between hydrocarbon residues of SDS [26]. Namely, above 0.6 CMC the SDS micelle is large enough to absorb larger amounts of resveratrol molecules, thus disturbing the spherical symmetry of the micelle. In that respect, the space between alkile residues of SDS filled with water molecules. These water molecules can form hydrogen bonds with resveratrol, which partly stabilizes the mixed micelle. NaCl, due to “salting-out effect,” removes the “Tanford water molecules” and disturbs the compactness, that is, stability of the mixed micelle.

Urea increases resveratrol solubilization capacity of sodium dodecyl sulfate within the entire concentration range analyzed. This can be explained by the fact that amino group of urea forms hydrogen bonds with sulphate ions of SDS at the outer surface of the micelle (urea molecule is located between the adjacent sulphate ions –O–SO_2_–O^−^ *⋯* **H–NH–CO–NH–H** *⋯* ^−^O–SO_2_–O–), which decreases the repulsive interaction between negatively charged polar heads.

### 3.2. The Fluorescence Study of Resueratrol in Mixed Micelles

If resveratrol molecule would not be incorporated in the internal cage of the micelle rate between emission intensity of the system resveratrol water solution and surfactant, *I* (resveratrol + surfactant), and intensity of emission of resveratrol water solution itself, *I* (resveratrol), would be one (analyzed surfactants do not emit on 360 nm).

Tables 1 and 2 show that resveratrol has differente microenvironment in mixed micelle depending on the nature of the applied surfactant (building unit).

In presence of natrium dodecyl sulfate (SDS) emission increases compared to the emission of pure resveratrol in water (Table 1). In concentration of 0.5 CMC rate between emission intensity of resveratrol in presence of aliphatic surfactant natrium dodecyl sulfate and emission of pure resveratrol is high. This means that resveratrol enters the hydrophobic microenvironment and hydrophobic interactions between aromatic ring and hydrophobic side chain of sodium dodecyl sulfate are formed and micelles are formed even in submicellar zone. It is known that formation of mixed micelle lowers the CMC value of surfactant which is the building unit of the examined micelle. With increase in temperature relation between intensity of resveratrol decreases since internal mobility of the SDS hydrophobic tail leads to partial hydration of the hydrophobic domain of the mixed micelle; that is, microenvironment of aromatic part of resveratrol is less hydrophobic. Increasement of the concentration of SDS monomer leads to rise in the size of the micelle since electrostatic repulsion is lower between several bigger micelles than between higher number of smaller micelles [27]. However, relation between intensities of resveratrol emission with higher concentration of SDS decreases. This is probably the consequence of the fact that one OH group of resveratrol forms hydrogen bond with sulphate group of SDS on the surface of the micelle and stabilises the micelle, that is, decreases internal mobility of SDS and possibility for hydration of the hydrophobic domain. However, with the rise in the size of the mixed micelle (increase in concentration of SDS) number of hydrogen bond between OH group of resveratrol and SDS per unit area of the aggregate decreases as well as stabilisation effect.

**Figure 6:**
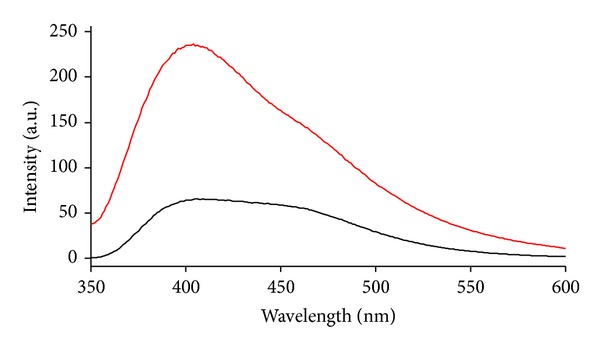
Emission of resveratrol (red line) and resveratrol in presence of S7-OD (black line).

**Figure 7:**
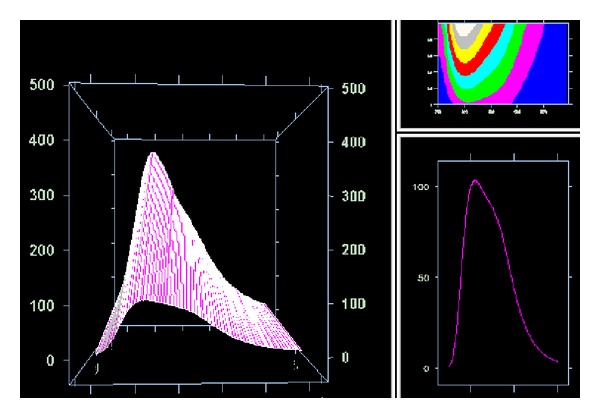
Emission spectra of pure resveratrol and resveratrol in presence of S7-OD on different temperatures (temperature increases from the observer to the image depth).

Rate intensity between intensity of emission of resveratrol in presence of surfactant S7-OD and resveratrol in water suggests that regiochemical recognition between resveratrol and micelle of S7-OD by hydrogen bond happens (Table 2, Figure 6). Hydrogen bond is formed between 7-oxo group and appropriate hydroxyl group of resveratrol (the effect of speed). Such hydrogen bond is formed entropically more favourable then hydrogen bond between water molecules themselves because oxo group is located in the micelle (there is no loss of entropy, that is, translator degree of movement). Fixing of resveratrol molecule thus reduces vibrational motion of resveratrol which would happen in hydrophobic environment as interior of micelle. Since this relation happens even in concentration of 0.5 CMC, formation of mixed micelle is confirmed so value of critical micelle concentration decreases. With higher temperatures micelle becomes more mobile and hydrogen bond is formed less efficiently (Figure 7). Increase in concentration of bile acid sodium salt has a small impact on the change of the rate of resveratrol emission since the size (aggregation number) of the bile acid micelle does not change a lot with the growth of the monomers comparing to SDS micelle [28].

## 4. Conclusion

In the resveratrol solubilization, sodium 3*α*,12*α*-dihydroxy-7-oxo-5*β*-cholanoate and sodium dodecyl sulfate exhibit different behavior. The investigated oxo derivative of cholic acid shows 3-fold higher solubilization capacity. Up to 1.4 CMC, the NaCl increases resveratrol solubilization of 3*α*,12*α*-dihydroxy-7-oxo-5*β*-cholanoate, whereas urea decreases solubilization capacity within the entire range, particularly beyond CMC.

NaCl slightly increases resveratrol solubilization capacity of sodium dodecyl sulfate at max. 0.6 CMC, exhibiting the contrary effect at higher CMC values. Urea increases solubilization capacity of SDA within the entire concentration range.

In presence of SDS resveratrol enters the hydrophobic microenvironment and hydrophobic interactions between aromatic ring and hydrophobic side chain of sodium dodecyl sulfate are formed and micelles are formed even in submicellar zone. Also entropically more favourable hydrogen bond is formed between 7-oxo group of S7-OD and appropriate hydroxyl group of resveratrol. Fixing of resveratrol molecule reduces vibrational motion of resveratrol which would happen in hydrophobic environment as interior of micelle. Since this relation happens even in concentration of 0.5 CMC, formation of mixed micelle is confirmed so value of critical micelle concentration decreases.

## Figures and Tables

**Table 1:** Intensity ratio between emission of resveratrol in presence of SDS and resveratrol in water.

SDS	*I*(resveratrol + SDS)/*I*(resveratrol)		
Temperature	0.5 CMC	1 CMC	2 CMC
0	3.9077	1.8871	1.2513
10	3.2406	1.2690	1.2863
20	2.9328	1.1431	1.6598
30	3.0573	1.1931	1.7912
40	2.8871	1.0520	1.5485
50	2.7602	1.0363	1.1486

**Table 2:** Intensity ratio between emission of resveratrol in presence of S7-OD and resveratrol in water.

S7-OD	*I*(resveratrol + S7-OD)/*I*(resveratrol)		
Temperature	0.5 CMC	1 CMC	2 CMC
0	0.4403	0.3430	0.4006
10	0.3351	0.2942	0.3015
20	0.2807	0.2437	0.2391
30	0.2916	0.2322	0.2766
40	0.2366	0.1831	0.2501
50	0.1876	0.1480	0.2090
